# Adjuvant Chemotherapy of Soft Tissue Sarcoma—Current Status

**DOI:** 10.1080/13577140020025850

**Published:** 2000-12

**Authors:** Ian Judson

**Affiliations:** Sarcoma Unit Royal Marsden Hospital Fulham Road London SW3 6JJ UK


*Sarcoma* (2000) **4**, 149–150

EDITORIAL

### Adjuvant chemotherapy of soft tissue sarcoma—current status

IAN JUDSON

*Sarcoma Unit, Royal Marsden Hospital, London, UK*

It is acknowledged that, although improvements can be made in local control, e.g. by the use of adjuvant radiotherapy,[1] the major challenge in the management of adult soft tissue sarcomas is the treatment of distant metastatic disease. We lack very effective drugs capable of providing long-term disease control once this event has occurred. Palliation is possible with doxorubicin, ifosfamide or combinations of these agents. However, although more intensive treatment may increase the response rate, long-term disease control is rare.[2] Randomised trials have failed to show a benefit for drug combinations over single-agent doxorubicin in terms of progression-free or overall survival.[3]

Prevention of distant relapse would represent a much better strategy if this were possible. Few randomised trials have demonstrated a survival advantage for adjuvant chemotherapy, although the largest single study did show improved local control in some situations.[4] A meta-analysis of published trials initially reported a survival advantage in favour of chemotherapy;[5] however, a subsequent study of the individual patient data, while demonstrating a 10% improvement in relapse-free survival, failed to prove a survival advantage.[6] There has been much debate concerning these data; in particular, the fact that many trials included patients with any grade of tumour and also tumours at unfavourable sites, e.g. uterus. There was a clear impression that patients with extremity tumours were more likely to benefit from chemotherapy.

A small study has been conducted by the Italian Sarcoma Group, in which only patients with high-grade (grade III/IV), large (>5 cm) or recurrent, extremity or limb girdle tumours were included. The treatment comprised an aggressive combination of high-dose ifosfamide and epirubicin, supported by granulocyte-colony stimulating factor (G-CSF). This study was closed in 1996 as a result of an interim analysis that demonstrated a survival advantage. At the time of the latest reported follow-up (1999), that survival advantage was maintained.[7] This is a small study in a selected group, with only 104 patients randomised. However, the results are consistent with the findings of the meta-analysis.

The validity of the meta-analysis has also been questioned for its inclusion of studies using single-agent doxorubicin and the generally low-dose intensity of the treatments used. The advocates of more aggressive treatment would cite the results of the Italian trial as evidence for the value of intensification. A small feasibility study of intensive adjuvant chemotherapy with ifosfamide, dacarbazine and doxorubicin, together with hyperfractionated accelerated radiotherapy, from the Austrian Sarcoma Study Group is reported in this issue.[8] This may represent effective treatment, especially for those patients with the highest risk of disease recurrence based on grade and tumour size. However, the study was extremely small and, as such, is of limited value. It is acknowledged in the report that studies such as this cannot be used as a guide to treatment.

A literature search for recent papers on adjuvant chemotherapy in sarcoma revealed 247 reports on adjuvant treatments but only four papers referred to randomised trials, two of which related to the meta-analysis and two were reviews. Verweij and Seynaeve[9] rightly emphasised the need to confine the use of adjuvant chemotherapy to the investigational setting. The European Organisation for Research and Treatment of Cancer Soft Tissue and Bone Sarcoma Group is currently conducting a prospective randomised trial of adjuvant chemotherapy. The study design addresses some of the criticisms of previous studies, by including quality control of histopathological diagnosis and by the early initiation of chemotherapy in those patients randomised to treatment. This study uses a haemopoietic growth factor to allow an increase in the dose of doxorubicin to 75 mg/m$^2$, but the dose of ifosfamide, at 5 g/m$^2$, is modest. Perhaps, also increasing the dose of ifosfamide is valuable in the adjuvant setting, as reported by Frustaci *et al.*[7] and Brodowicz *et al.*[8] The challenge lies in being able to demonstrate the true value of such aggressive approaches in prospective randomised trials of sufficient size to yield meaningful results.

Correspondence to: Ian Judson, Sarcoma Unit, Royal Marsden Hospital, Fulham Road, London SW3 6JJ, UK

1357-714X print/1369-1643 online/00/040149-02 © 2000 Taylor & Francis Ltd

*DOI: 10.1080/13577140020025850*

### References

1 Yang JC, Chang AE, Baker AR, *et al.* Randomized prospective study of the benefit of adjuvant radiation therapy in the treatment of soft tissue sarcomas of the extremity. *J Clin Oncol* 1998; 16:197–203.
2 Blay J-Y, van Glabbeke M, Nielsen OS, *et al.* Five-year survivors in patients (pts) with advanced soft tissue sarcoma (ASTS) treated with doxorubicin: a study in 1742 patients (STBSG) [abstract 1973]. *Proc Am Soc Clin Oncol* 1998; 17:512a.
3 Santoro A, Tursz T, Mouridsen H, *et al.* Doxorubicin versus CYVADIC versus doxorubicin plus ifosfamide in first-line treatment of advanced soft tissue sarcomas: a randomized study of the European Organization for Research and Treatment of Cancer Soft Tissue and Bone Sarcoma Group. *J Clin Oncol* 1995; 13:1537–45.
4 Bramwell V, Rouesse J, Steward W, *et al.* Adjuvant CYVADIC chemotherapy for adult soft tissue sarcoma—reduced local recurrence but no improvement in survival: a study of the European Organization for Research and Treatment of Cancer Soft Tissue and Bone Sarcoma Group. *J Clin Oncol* 1994; 12:1137–49.
5 Tierney JF, Mosseri V, Stewart LA, *et al.* Adjuvant chemotherapy for soft-tissue sarcoma: review and meta-analysis of the published results of randomised clinical trials. *Br J Cancer* 1995; 72:469–75.
6 Sarcoma Meta-Analysis Collaboration. Adjuvant chemotherapy for localised resectable soft-tissue sarcoma of adults: meta-analysis of individual data. *Lancet* 1997; 350:1647–54.
7 Frustaci F, Gherlinzoni F, Paoli A, *et al.* Maintenance of efficacy of adjuvant chemotherapy (CT) in soft tissue sarcomas (STS) of the extremities up-date of a randomized trial [abstract 2108]. *Proc Am Soc Clin Oncol* 1999; 18:546a.
8 Brodowicz T, Schwameis E, Widder J, *et al.* Intensified adjuvant *IFADIC* chemotherapy for adult soft tissue sarcoma: a prospective randomized feasibility trial. *Sarcoma* 2000; 4:151.
9 Verweij J, Seynaeve C. The reason for confining the use of adjuvant chemotherapy in soft tissue sarcoma to the investigational setting. *Semin Radiat Oncol* 1999; 9:352–9.
